# Urban poor are the most endangered by socio-natural hazards, but not exclusively: the 2025 Granizal Landslide case

**DOI:** 10.1007/s10346-025-02680-y

**Published:** 2025-12-19

**Authors:** Ugur Ozturk, Anika Braun, Juan Camilo Gómez-Zapata, Edier Aristizábal

**Affiliations:** 1 Department of Geography and Regional Research, University of Vienna, Vienna, 1010 Austria; 2 Section 2.6-Seismic Hazard and Risk Dynamics, GFZ Helmhotz Centre for Geosciences, Potsdam, 14473 Germany; 3 Department of Engineering Geology, Technische Universität Berlin, Berlin, 10587 Germany; 4 Climate Analytics, Berlin, 10969 Germany; 5 Departamento de Geociencias y Medio Ambiente, Universidad Nacional de Colombia, Medellín, 65-223 Colombia

**Keywords:** Granizal Landslide, Climate change, Socio-spatial segregation, Municipal capacity, Informal settlements, Medellín

## Abstract

In June 2025, the rainfall-induced Granizal Landslide claimed 27 lives across two jurisdictions in the Medellín metropolitan area. Based on our empirical analyses of a local socio-economic index, we argue that socio-spatial segregation may have increased the likelihood and impact of the disaster. The event’s location also underscores the challenges faced during emergency response and post-disaster recovery across administrative boundaries, particularly given the stark contrast in the capacities of municipal authorities. We suggest that intensifying climate change may lead to events that exceed current engineering design criteria, thereby exposing even formal neighborhoods—often considered more physically resilient—to substantially elevated risk levels and narrowing the gap with informal settlements.

## Introduction

**Fig. 1:**
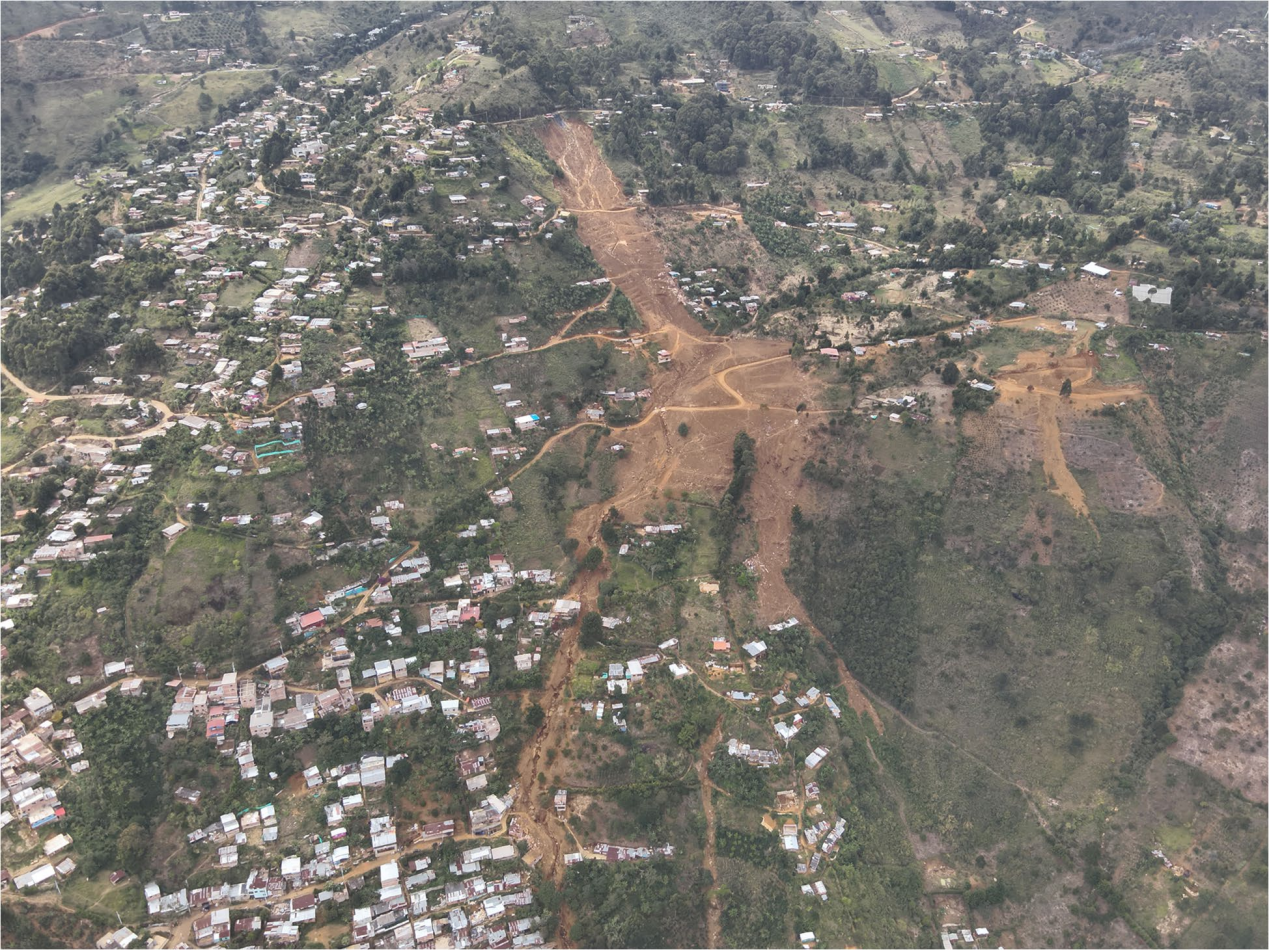
Image of Granizal Landslide near Bello with a drone from the opposite side (facing east). The drone image was taken on July 28, 2025, by Departamento Administrativo de Gestión del Riesgo (DAGRD)

On June 24, 2025, after 3 am, a rainfall-induced landslide buried approximately 50 houses, killing 27 inhabitants, and displacing over 1000 people (Fig. 1). The landslide occurred in a hillside sector informally known as *Granizal*, extending across the boundary between two municipalities within the Medellín metropolitan area. While the landslide crown was located in Bello, the toe reached rural neighborhoods within Medellín. The primary trigger was continuous rainfall that lasted for 1.5 days before the Granizal Landslide.

Rainfall-triggered landslides in humid tropical mountains are highly sensitive to changes in the intensity, duration, and sequencing of precipitation events. Anthropogenic climate change is projected to increase the frequency and intensity of heavy precipitation in several tropical regions, including the northern Andes (IPCC 2021; Pabón-Caicedo et al. 2020). These projections may be interpreted as increased exceedance of pore-pressure thresholds in shallow soils, shorter recovery times between rainstorms, and a greater likelihood of landslide clusters rather than isolated failures, leading, overall, to higher rates of rainfall-induced landsliding (Gariano and Guzzetti 2016).

Although climate change is undoubtedly a major contributor, the Granizal disaster is yet another example of the high human toll on the most vulnerable population, who are pushed to live in landslide-prone areas (Ozturk et al. 2022; Bastos Moroz & Thieken 2024). As urban populations grow, cities’ margins expand towards steeper hillslopes, especially in emerging tropical countries, exposing more people to landslide hazards (Ferrer et al. 2025) . Without sufficient resources and institutional support, informal settlers are forced to manage their own infrastructure and housing. Self-built informal construction often lacks formal planning and infrastructure (Henderson 2002), which can increase landslide potential, for example, through inadequate water management (Bozzolan et al. 2020). After all, increasing rainfall rates would continue to pressure any water management structure, regardless of its formality.

Motivated by the Granizal Landslide, we empirically assessed the socio-spatial segregation in Medellín. In the “Results” section, we show that socio-spatial segregation may be exposing the urban poor disproportionately to landslide hazards in the city. However, a considerable portion of wealthier neighborhoods is also located on steep terrain in Medellín. We discuss that these wealthier, more resilient neighborhoods may be increasingly exposed to landslides as climate change intensifies.

## Study area

**Fig. 2:**
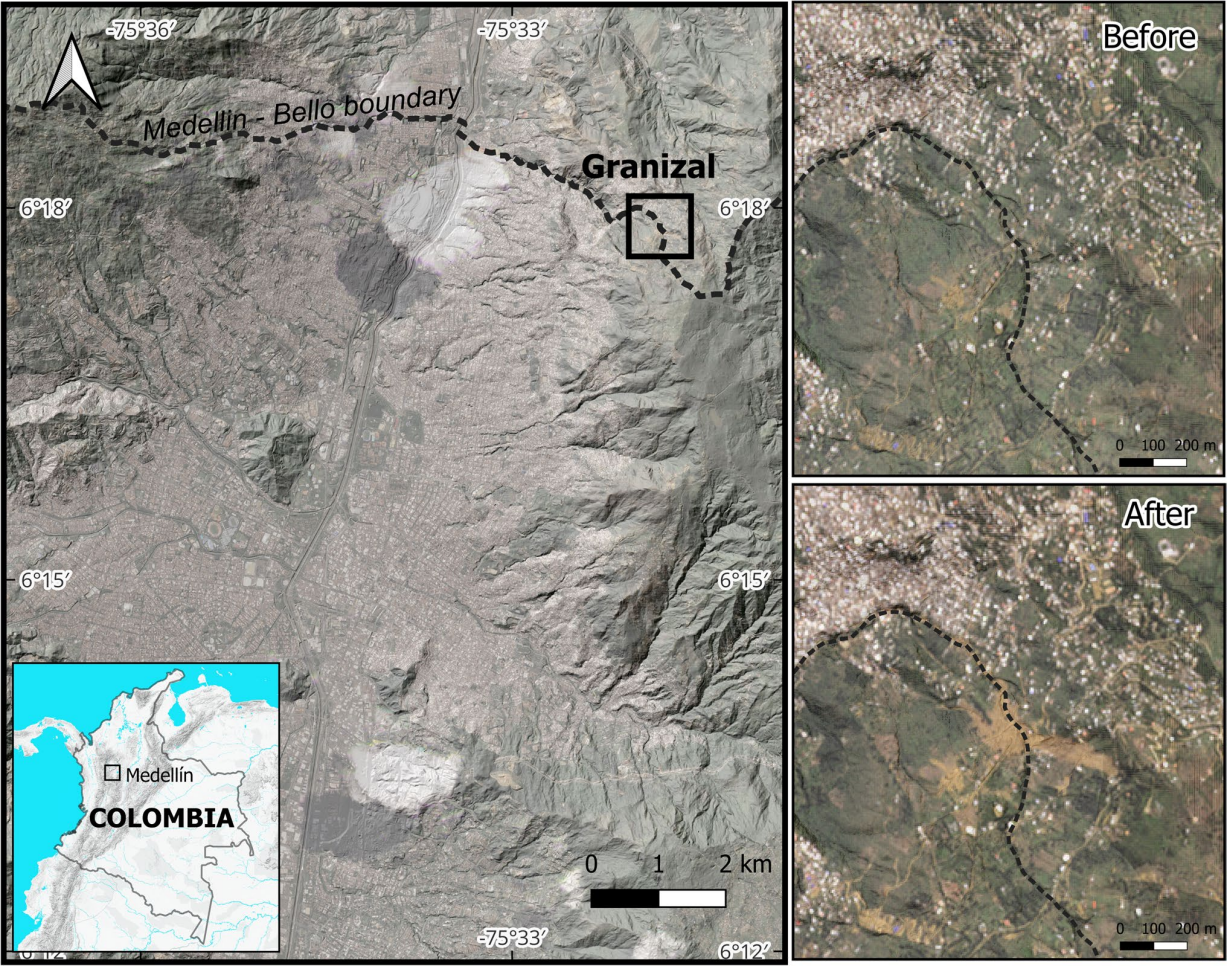
Location of the Granizal Landslide in Medellín, Colombia, with before and after the event images (Image©2025 Planet Labs PBC 2025)

**Fig. 3:**
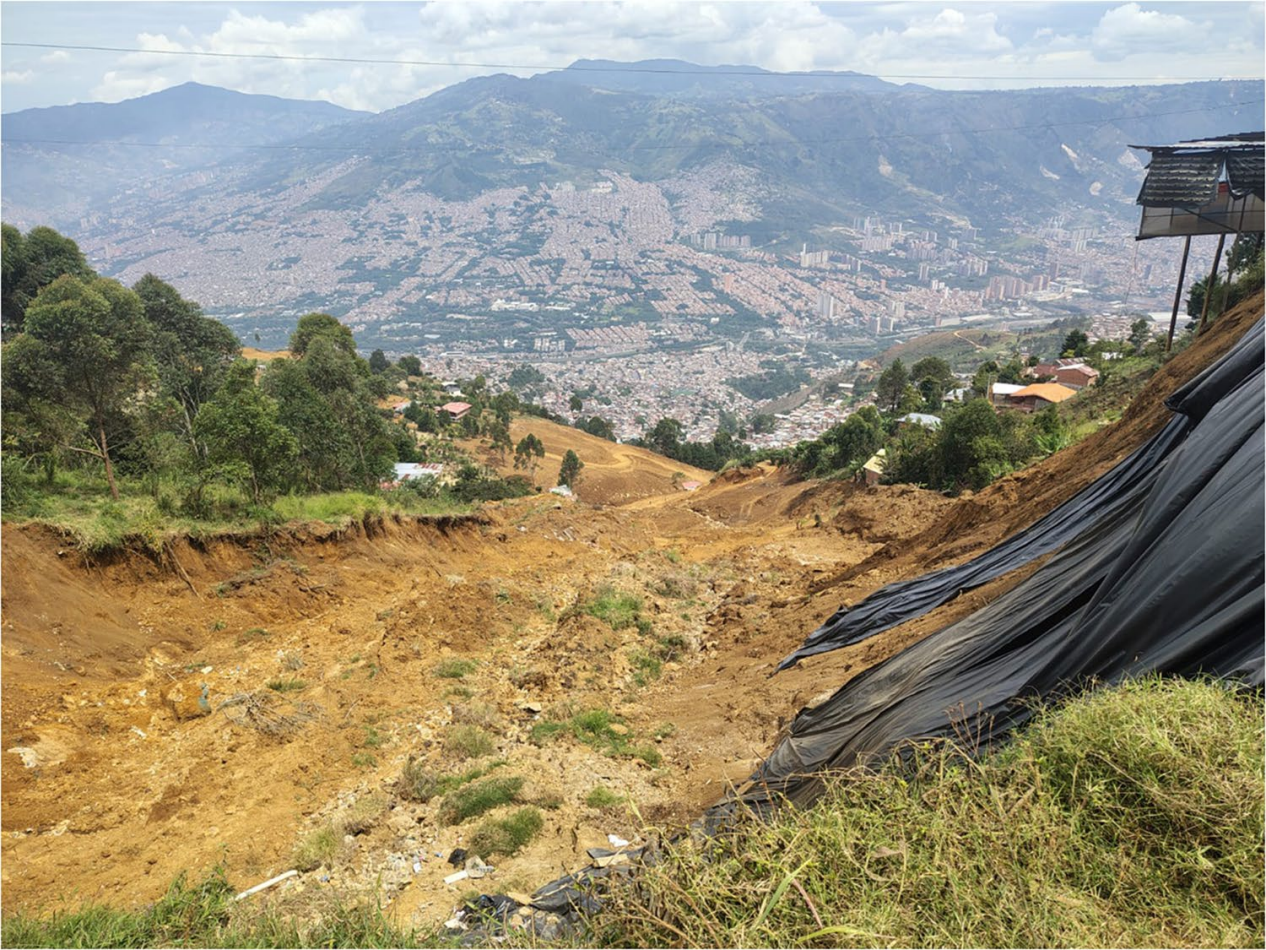
Image of Granizal Landslide near Bello taken from above the head scarp (facing west). Medellín is visible in the background. The image was taken on July 28, 2025, by Departamento Administrativo de Gestión del Riesgo (DAGRD)

The metropolitan area of Medellín lies in the narrow Aburrá Valley, forcing housing onto the surrounding hillslopes (Fig. 2). The valley broadens only slightly where the urban core has taken root (visible in the background of Fig. 3), forcing most housing to climb the surrounding hillslopes. This topographic constraint and associated landslide danger have produced a socio-spatial segregation. Hillslope steepness increases towards poorer settlements along with elevated landslide hazards and impacts (Nieto et al. 2025).

The complex Granizal Landslide initiated as a translational slide and evolved into a flow. The failure occurred in saprolite soil developed over the Medellín Dunite, an ultrabasic igneous rock unit (Fig. 3). The source volume was estimated at 75,000 m^3^, with a failure surface measuring 143 m in length and 50 m in width. A major contributing factor appears to be the local hydrogeology, as field inspections identified multiple water seeps and springs emerging directly from the landslide’s main scarp, suggesting complex groundwater behavior within the dunite weathered rock profile. A failure in the sewage system may have caused additional rainwater to flow onto the unstable hillslope. However, this speculative hypothesis has not yet been confirmed and will be evaluated in a forthcoming technical investigation.

## Data and methods

To characterize the socio-spatial configuration of exposure in the Aburrá Valley (including Medellín and Bello), we used a residential building exposure model developed by the Universidad EAFIT in collaboration with the local cadastral authorities and the GEM (Global Earthquake Model) foundation. The underlying data have been previously used in local physical risk analyses (e.g., Acevedo et al. 2017, 2020). This dataset integrates demographic and building information at the neighborhood level, including the Strata (utility tax category, defined based on wealth), estimates of the number of dwellings, residents, and replacement values for residential assets. These variables provide a spatially explicit approximation of wealth and exposure across 357 neighborhoods.

We linked these neighborhood indicators with the corresponding hillslope gradients in a grid derived from a 30-m Shuttle Radar Topography Mission (SRTM) (NASA 2013) digital elevation model using the Zevenbergen and Thorne (1987) method. Built-up areas were delineated from the European Space Agency (ESA) global land-cover product (MRLC v2.0.7; Harper et al. 2023) to ensure hillslope angle estimates corresponded to developed terrain only. The processing required superimposing the datasets, which we accomplished using the nearest-neighbor method (Schwanghart and Scherler 2014). This combination allowed us to compare the spatial distribution of wealth and exposure with topographic steepness across socio-economic categories.

In our analyses, we first examine landslide locations across hillslope and elevation gradients. For this, we calculated the distribution of hillslope angles and elevation for the entire Medellín metropolitan area, including the municipalities of Medellín and Bello, as well as Envigado, Itagüí, Sabaneta, La Estrella, Caldas, Copacabana, Girardota, and Barbosa. However, when assessing socio-spatial segregation, we restrict our analysis to the municipality of Medellín, as the Strata (utility tax category) data required for this component are only available for this administrative area.

## Results

**Fig. 4:**
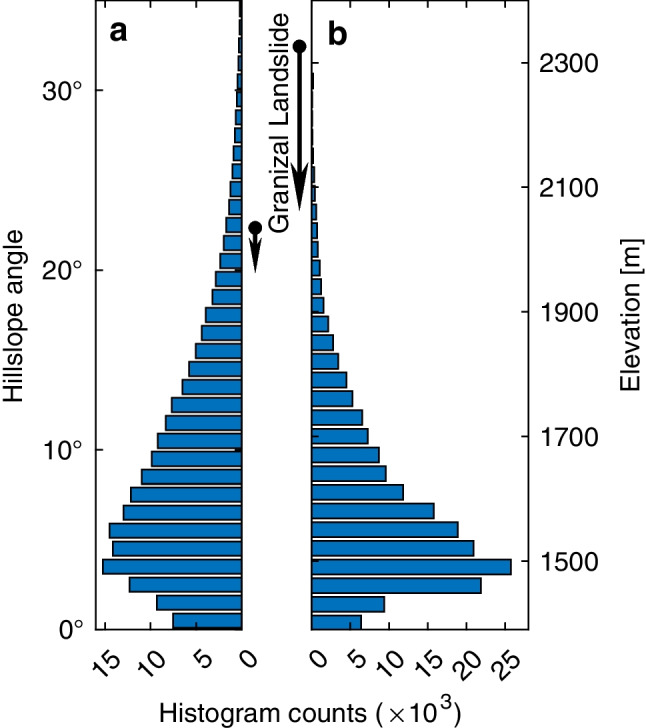
Hillslope angle (**a**) and elevation (**b**) distribution of the Medellín metropolitan area (covering both Bello and Medellín municipalities). Maximum elevation (hillslope angle) is 2557 m (71°), and minimum is 1313 m (0°). Arrows in between the subplots show the Granizal Landslide’s range from crown to toe

We first located the Granizal Landslide within the hillslope angle and elevation range of the Medellín metropolitan area (covering both Bello and Medellín municipalities). The Granizal Landslide occurred on hillslopes of 20–22°, which is among the steepest terrain in the metropolitan area (Fig. 4a). The landslide moved from above 2300 m to under 2100 m (Fig. 4b). These elevations are the highest in the area, which covers the range from about 1400 m to 2400 m. The majority of the metropolitan area is located at an elevation below 2000 m. These observations may indicate that the metropolitan area is gradually entering very steep, landslide-prone terrain and is becoming increasingly subject to landslide disasters.

**Fig. 5:**
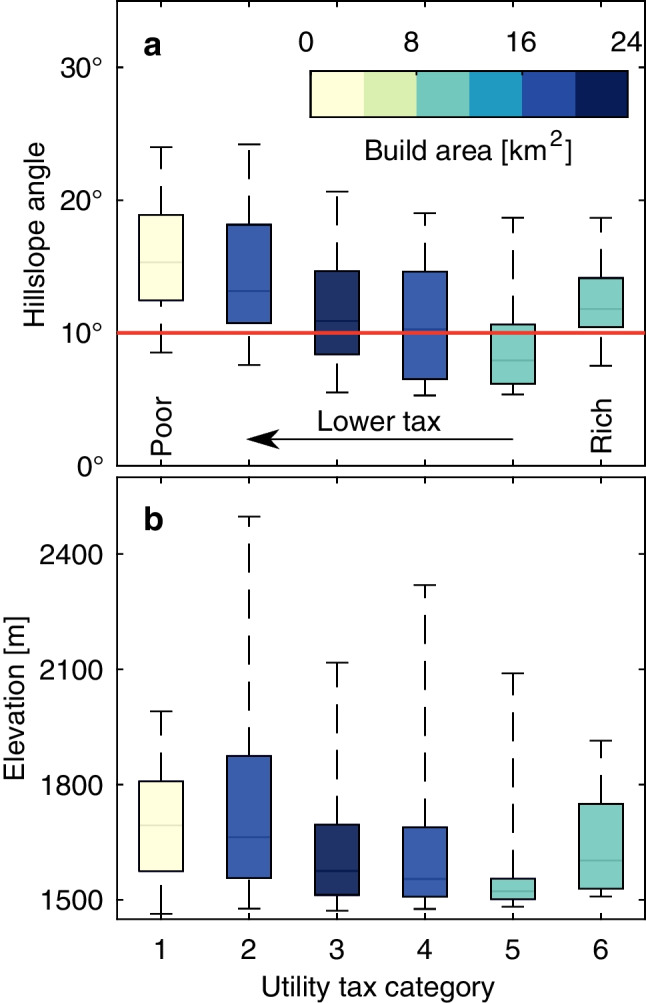
Hillslope angle (**a**) and elevation (**b**) of the buildup area in Medellín, categorized by utility tax, known as Strata, which determines the socio-economic status of different neighborhoods. For example, the utility tax decreases as the categories get lower. Hillslope angle increases generally towards poorer categories

Our first-order approximation shows that in Medellín, the mean hillslope distribution per category increases toward low-tax neighborhoods, except for the highest-taxed (i.e., tax category 6; Fig. 5a). In contrast, when considering elevation alone, no clear pattern emerges, except for a rise at the extreme upper end of the distributions (Fig. 5b). Most low-income residents (i.e., tax categories 1–3) live in informal housing on the steep northern hillslopes. Conversely, the equally steep southern hillslopes host better-engineered high-income developments (i.e., tax category 6), such as gated communities. Thus, the richest and poorest are similarly exposed to landslide hazards but with starkly contrasting vulnerabilities, revealing the city’s socio-economic segregation.

In addition to having generally steeper hillslope distributions, poorer neighborhoods also occupy at least twice the area on those steeper hillslopes, when compared to the highest category (compare tax categories 2 and 6 in Fig. 5a). Hence, the central issue of poor people being more exposed to landslides is undoubtedly there. However, landslide risk is not limited to informal settlements. Cities might systematically push the formal and informal populations into landslide-prone areas, especially when construction space is limited, as in Medellín.

## Discussion

The landslide fatality record of Medellín underscores the consequences of the city’s spatial configuration, and associated building practices and coping capacity. Past landslide disasters (GeoHazards database, Aristizábal et al. 2025) have killed around 800 people in Medellín since the 1950s. However, only 16 of these fatalities occurred in southern elevated settlements. To quantify the link between urbanization and landslide occurrences (e.g., Aristizábal & Sánchez 2020; Raška et al. 2023), recent advances in mechanistic modeling have shown that it can provide quantitative information on the dynamic interactions between forms of urbanization and landslides (Bozzolan et al. 2020, 2023). Such models also have the potential to integrate climate projections (e.g., Jimenez et al. 2025). These advances may offer quantitative insights into future hazards and early guidance for participatory and equitable urban design (e.g., Klimeš et al. 2019).

Event-attribution work now links a share of heavy-precipitation extremes and associated landslide disasters to anthropogenic greenhouse gas emissions, including a deadly landslide event in southeastern Brazil, where climate change increased both the intensity and the probability of the triggering rainfall (Barbosa et al. 2025). For the Andes, detection-and-attribution analysis further indicates that anthropogenic climate change already contributes to observed hydroclimate-related impacts, including floods and landslide-prone conditions (Ochoa-Sánchez et al. 2025). In rapidly urbanizing tropical catchments, including the Medellín metropolitan area, these climatic trends superimpose on settlement expansion onto steep hillslopes, implying that events comparable to the 2025 Granizal Landslide may occur under increasingly adverse combinations of rainfall forcing and exposure. After all, rainfall patterns and landslide occurrences are closely linked in Colombia (Vega et al. 2024).

Rainfall, increasingly altered by climate change, is the primary control on landslide occurrence in the tropics (e.g., Jakob 2022; De Oliveira Andrades-Filho et al. 2025). For example, in Colombia, rainfall is responsible for roughly 90% of all landslides (Aristizábal & Sánchez 2020). However, future urbanization will likely determine the number of fatal landslides (e.g., Alcántara-Ayala 2025). In addition to exposing disadvantaged informal neighborhoods, projected increases in rainfall extremes may also challenge the perceived safety of wealthier areas. This shift may require revisiting the hazard assessment of planned infrastructure.

Parameters used to design the planned infrastructure often rely on historical data, which may soon be outdated. Although not in the same place, a striking example here is the active landslide in Rancho Palos Verdes, the USA (Paddison 2024). To reduce landslide movement, pumping infrastructure was installed in the 1980 s and 1990 s to dewater the landslide body. However, water pressure is increasing due to climate change, requiring ongoing infrastructure investments and elevating management costs (Toohey 2025). The extent to which these evolving hazards translate into future losses will depend on local adaptation efforts and on the adaptive capacity of institutions and communities, including their ability to upgrade drainage and slope-stabilization works, manage urban growth on steep hillslopes, and strengthen early warning and preparedness. Hence, the presumed resilience of high-income neighborhoods could erode with time due to climate change, increasing both human and economic risks.

The 2025 Granizal Landslide also underscores a critical yet often overlooked challenge: disasters that transcend municipal boundaries. The landslide crown was located in the municipality of Bello. Yet, the landslide toe reached the adjacent municipality of Medellín, causing fatalities and damage in both jurisdictions. While emergency response efforts were rapidly coordinated—shelters were established in both municipalities and relief agencies collaborated effectively—the unequal institutional capacities soon became evident. Medellín, with greater financial and logistical resources, assumed a leading role in relief and support, prompting many affected families from Bello to seek assistance there. However, long-term recovery and risk reduction efforts have proven more challenging to coordinate in practice due to legal, administrative, and political fragmentation. As urban areas continue to merge into larger metropolitan regions, institutional frameworks must evolve to manage shared socio-natural hazards that ignore political boundaries. This case illustrates how metropolitan areas must adapt their institutional frameworks to manage socio-natural hazards that disregard municipal boundaries.

## Conclusion

In our manuscript, we provide a brief overview of the Granizal Landslide. Using this example, our main point is to highlight landslide occurrences and potential in informal settlements, which may be exacerbated by socio-spatial segregation in Medellín, as we show in our results. Although informal districts should be the prime focus of future modeling solutions, we should not forget that climate change is gradually intensifying and may soon render the design criteria used for planning formal neighborhoods obsolete. Hence, our concluding message is that future rainfall changes may also lead to catastrophic landslide impacts in formally planned urban neighborhoods, challenging the assumption that only informal settlements are at high risk.

## Data Availability

All the data used in this manuscript are open-access. They can be accessed via the source articles cited in the respective location.
